# Distinct synaptic mechanisms drive the behavioral response to acute stress and rapid correction by ketamine

**DOI:** 10.1038/s41386-024-01908-0

**Published:** 2024-07-01

**Authors:** Ji-Woon Kim, Benjamin Kleinfelter, Ege T. Kavalali, Lisa M. Monteggia

**Affiliations:** 1 Department of Pharmacology, School of Medicine, Vanderbilt University, Nashville, TN 37240 USA; 2 College of Pharmacy, Kyung Hee University, Seoul, Republic of Korea; 3 Department of Regulatory Science, Graduate School, Kyung Hee University, Seoul, Republic of Korea; 4 Institute of Regulatory innovation through Science, Kyung Hee University, Seoul, Republic of Korea

**Keywords:** Emotion, Cellular neuroscience

## Abstract

Prevailing hypotheses on the mechanisms of antidepressant action posit that antidepressants directly counteract deficiencies in major neurotransmitter signaling systems that underlie depression. The rapidly acting antidepressant ketamine has been postulated to correct excess glutamatergic signaling via glutamatergic antagonism leading to the rescue of neuronal structural deficits and reversal of behavioral symptoms. We studied this premise using systemic administration of the acetylcholinesterase inhibitor physostigmine, which has been shown to rapidly elicit a shorter-term period of depressed mood in humans via cholinergic mechanisms. We observed that physostigmine induces acute stress in tandem with long term depression of glutamate release in the hippocampus of mice. However, ketamine rapidly acts to re-establish glutamatergic synaptic efficacy via postsynaptic signaling and behaviorally masks the reduction in passive coping induced by physostigmine. These results underscore the divergence of synaptic signaling mechanisms underlying mood changes and antidepressant action and highlight how distinct synaptic mechanisms may underlie neuropsychiatric disorders versus their treatment.

## Introduction

Major depressive disorder (MDD) is a heterogeneous mental disorder in which patients may present with a variety of symptoms. Traditional antidepressants are widely prescribed and target the monoaminergic system to produce therapeutic effects to many patients. These findings led to the monoamine hypothesis of depression which suggests that depletion of serotonin, norepinephrine, and/or dopamine levels in the central nervous system leads to depressive symptoms and in turn, antidepressants counter these deficiencies via blockade of reuptake or degradation of these monoaminergic neurotransmitters [1]. While this parsimonious premise has been repeatedly challenged in the case of monoaminergic transmission, the same reasoning has been used to explain the antidepressant action of ketamine [2]. Specifically, prolonged stress and depression have been associated with a sustained increase in extracellular glutamate and glutamatergic signaling, as excess glutamate precipitates excitotoxicity, altered synaptic strength, reduced dendritic spine density, dendritic retraction, and reduced dendritic branching in the prefrontal cortex and hippocampus [3]. Ketamine, via blockade of NMDA receptors, is thought to rapidly counter these pathological changes by directly targeting and alleviating the consequences of this putative excitotoxic insult. However, the validity of whether ketamine produces its antidepressant effects by correcting deficits in neurotransmission has not been explicitly evaluated.

Physostigmine is a short-acting, reversible acetylcholinesterase inhibitor that rapidly induces a shorter-term period of depressed mood in healthy subjects as well as patients with mood disorders, typically within 20 min, and lasting for approximately an hour [4–6]. Physostigmine can produce passive coping responses in the forced swim test in mice suggesting increased cholinergic tone, which has been localized to the hippocampus [7, 8]. Taking advantage of physostigmine’s rapid induction of passive coping responses, we probed the overlap between mechanisms underlying physostigmine-induced behavioral effects and its alleviation by ketamine. To investigate this premise, we analyzed the molecular mechanisms underlying physostigmine-induced synaptic effects in the hippocampal Schaffer collateral pathway, as well as the effects of ketamine on the synaptic and behavioral alterations induced by physostigmine.

## Materials and methods

### Animals

This study utilized C57BL/6J male mice, aged 2–4 months. To create the conditional *Trkb* KO mice, *Trkb*^fl/fl^ mice [9] were crossed with *Camk2a*-Cre93 mice lines [10]. For the electrophysiology studies, both male *Trkb* KO mice and their littermate controls, aged 2–3 months, were included. The animals were kept under a 12-h light/12-h dark cycle, at an ambient temperature of 23 ± 3 °C, and humidity of 50 ± 20%, with free access to chow pellets and water. All animal procedures adhered to the guidelines for the care and use of laboratory animals and were approved by the Institutional Animal Care and Use Committee (IACUC) at Vanderbilt University.

### Drug treatment

Physostigmine salicylate (Cat No. 1537003) and ketamine hydrochloride (500 mg/5 ml) were obtained from Sigma Aldrich and Pfizer, respectively. Physostigmine (0.25 mg/kg) and ketamine (5 mg/kg) were dissolved or diluted in the saline just before each behavioral test.

### Forced swim test

Forced swim test was conducted as described previously [11].After a 2-h habituation period in the testing room, the mice received an injection of physostigmine. Fifty minutes later, each mouse was placed in a 4 L Pyrex glass beaker containing 3 L of water at 23–24 °C for a total of 6 min. The immobility time of the mouse was recorded during the last 4 min of the test. Water was changed between each mouse. To investigate the impact of ketamine on the behavioral changes induced by physostigmine, ketamine was administered 20 min after the physostigmine treatment.

### Locomotor activity test

After spending 2 h in the testing room to acclimate, the mice received an injection of physostigmine. After 50 min, the mice were transferred to standard cages with red light, and their locomotor activity monitored for 60 min using photocell beams connected to computer acquisition software (San Diego Instruments). For the ketamine treatment experiment, either ketamine or saline was given 20 min after the physostigmine treatment, and then their locomotor activity was measured. We used different cohorts for these experiments.

### Hippocampal slice preparations

Hippocampal slices were prepared as described previously [12]. Mice aged 2–4 months were anesthetized with isoflurane and then decapitated. The brain was immediately removed and sliced (thickness: 400 μm) using ice-cold dissection buffer with a vibratome (Leica VT 1000S). The dissection buffer contained the following components (in mM): 2.6 KCl, 1.25 NaH_2_PO_4_, 26 NaHCO_3_, 0.5 CaCl_2_, 5 MgCl_2_, 212 sucrose, and 10 *d*-glucose. To record field potentiation in the SC–CA1 synapses, the CA3 region was surgically removed from each slice after sectioning. The slices were then transferred to a reservoir chamber filled with ACSF (artificial cerebrospinal fluid) containing the following (in mM): 124 NaCl, 5 KCl, 1.25 NaH_2_PO_4_, 26 NaHCO_3_, 2 CaCl_2_, 2 MgCl_2_, and 10 D-glucose. The slices were incubated at 30 °C for a 3-h recovery period. Both ACSF and dissection buffer were equilibrated with 95% O_2_/5% CO_2_.

### Hippocampal field recording

Hippocampal field recording was conducted as done previously [13]. For the recording, slices were placed in a submerged recording chamber, maintained at 30 °C, and continuously perfused with ACSF at a rate of 3 ml/min. fEPSPs (field excitatory postsynaptic potentials) were recorded using extracellular recording electrodes filled with ACSF (resistance, 1–2–MΩ) and positioned in the CA1 area stratum radiatum. The fEPSPs were evoked by monophasic stimulation (duration 200 μs) of Schaffer collateral/commissural afferents using a concentric bipolar microelectrode (FHC). Stable baseline responses were recorded every 30 s using a stimulus that produced 50–75% of the maximum peak. The fEPSPs were filtered at 2 kHz and digitized at 10 kHz on a personal computer using customized software (LabVIEW, National Instruments). The response was measured as the initial slope (10–40% of the rising phase) of the fEPSPs. After 20 min of baseline measurement, physostigmine (10 μM) was perfused for 20 min to induce synaptic suppression. All responses were normalized to the baseline responses. The paired-pulse ratio (PPR) was assessed with interstimulus intervals of 20, 30, 50, 100, 200, 400, and 500 msec at designated time points. PPR was calculated as the slope ratio of the second to the first response (P2/P1). The input/output response was measured to determine the response intensity using 4, 8, 12, 16, 20, and 24 μA stimulation.

### AChE activity assay

Acetylcholinesterase activity was assayed by means of a colorimetric assay kit according to the manufacturer’s instructions (ab138871, abcam). Intact slices were incubated in the slice recording chamber. Slices were collected at designated time points. Collected slices were homogenized with phosphate buffered saline. The homogenates were centrifuged at 10,000 × *g* for 10 min, and supernatant was collected into a new tube. After making the mixture of acetylcholine-reaction buffer containing acetylthiocholine and 5,5’-dithio-bis-2-nitrobenzoic acid (DTNB) and the homogenate supernatant, the mixture was incubated for 30 min at room temperature. The absorbance of the mixture was monitored at 410 nm using POLARstar Omega plate reader (BMG Labtech).

### Statistical analysis

All statistical analyses were performed using GraphPad Prism 10 software. The normality of data was checked by the Shapiro–Wilk test. Homogeneity of variance was checked using Bartlett’s test. For comparison between two groups, two-tailed paired or unpaired t-test, and Welch’s correction t-test was performed. For comparisons of three groups, repeated measures (RM) one-way analysis of variance (ANOVA), one-way ANOVA, or Friedman test were performed. Tukey’s multiple comparisons test or Dunn’s test was used for post-hoc comparisons after the ANOVA tests. For detailed statistics information, see Supplementary Table 1. Statistical significance was designated as asterisks depending on *P*-value. *P < 0.05, **P < 0.01, ***P < 0.001.

## Results

### Physostigmine suppresses presynaptic glutamate release probability in the hippocampus

**Fig. 1:**
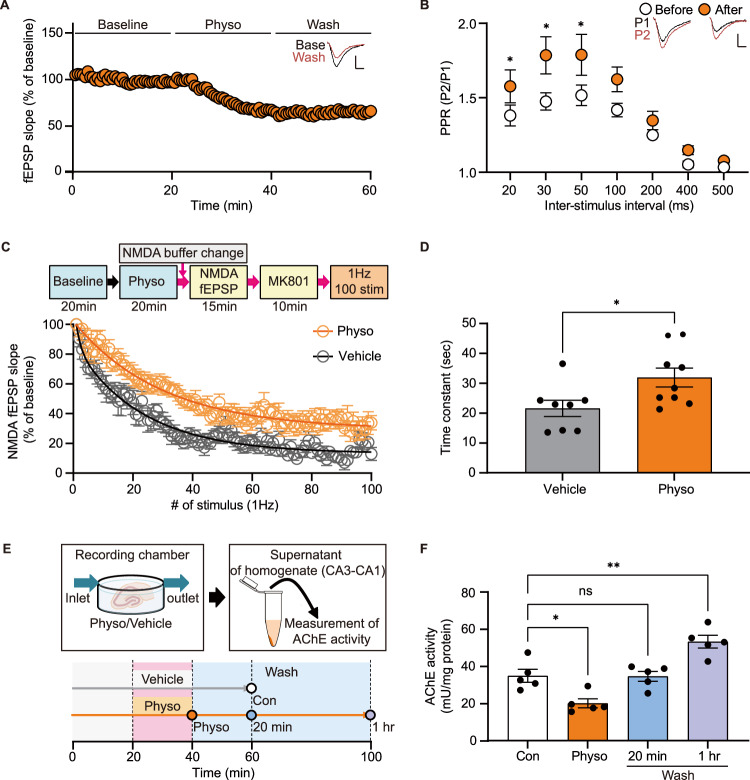
Physostigmine decreases presynaptic release in the hippocampal Schaffer collateral pathway. **A**, **B** fEPSPs and PPRs were recorded in the hippocampal Schaffer collateral pathway before and after perfusion of physostigmine onto the hippocampal slices. Physostigmine induces synaptic suppression (**A**) and increased PPRs (**B**) at interstimulus intervals of 20–100 msec (*n* = 5 slices from 2 mice). **C**, **D** To analyze the changes in presynaptic release probability following physostigmine treatment, we delivered 100 stimulations at a frequency of 1 Hz in the presence of MK801. Subsequently, NMDA fEPSPs were measured (vehicle, physo: *n* = 8, 9 slices from 4, 5 mice). **D** Time constant of the exponential decay of the NMDA fEPSPs was analyzed from **C**. Acetylcholinesterase activity was measured in the CA3-CA1 area of the hippocampal slices, as described in the schematic diagram (**E**), and the results are presented in **F** [*n* = 5 mice for each group]. Graphs represent mean ± S.E.M.; n.s. not significant, physo physostigmine. For detailed statistical information, see Supplementary Table 1.

To evaluate the impact of acute physostigmine administration on glutamatergic neurotransmission, we measured field excitatory postsynaptic potentials (fEPSPs) and paired pulse ratios (PPRs) before and after perfusion of physostigmine (Fig. 1A, B). PPR serves as a measure of presynaptic release probability, with increased PPRs suggesting a decrease in presynaptic release probability [14]. Consistent with previous findings [12, 15], we observed a decrease in fEPSPs within 20 min of physostigmine perfusion, with the suppressed synaptic response persisting for over 20 min (Fig. 1A). We also detected an increase in PPRs, specifically during the 20–50 msec inter-stimulus interval condition, providing further evidence that physostigmine suppresses presynaptic release probability (Fig. 1B).

To assess potential changes in neurotransmitter release probability by physostigmine, we analyzed the decay kinetics by measuring NMDA fEPSP in response to 100 stimulations delivered at 1 Hz in the presence of MK801. MK801 functions as a use-dependent NMDA receptor blocker, and its rapid blockade of NMDAR activity reflects the heightened frequency of NMDAR activation resulting from increased glutamate release [16]. Our findings revealed that physostigmine slowed the rate of NMDAR activity decay, indicating a reduction in presynaptic glutamate release probability (Fig. 1C, D).

The reduction in presynaptic glutamate release probability could be attributed to either continued inhibition of acetylcholinesterase activity or to prolonged signaling and synaptic changes. To explore these possibilities, we measured acetylcholinesterase activity in the hippocampal CA3-CA1 region following incubation of the slices in the same conditions used for the fEPSP recordings. We observed a significant decrease in acetylcholinesterase activity 20 min after physostigmine treatment, with this effect restored within 20 min after washing out the drug. Of note, after a 1-h washout period, acetylcholinesterase activity was significantly higher compared to levels in the control group, suggesting compensatory acetylcholinesterase expression (Fig. 1E, F). These findings suggest the suppressed presynaptic function observed following physostigmine treatment is not due to persistent alterations in acetylcholinesterase activity but rather arise from prolonged signaling and synaptic changes.

### Ca^2+^-dependent signaling mediates the decrease in glutamate release induced by physostigmine

**Fig. 2:**
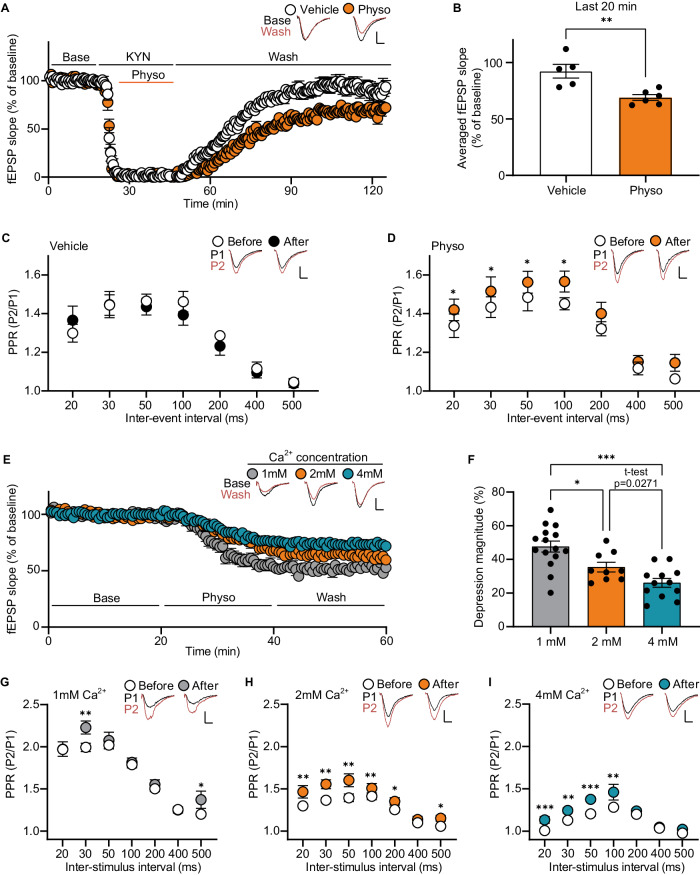
Physostigmine suppresses presynaptic release through a presynaptic Ca^2+^-dependent mechanism. **A**–**D** fEPSPs and PPRs were measured in the hippocampal Schaffer collateral pathway before and after co-treatment with kynurenic acid and physostigmine. To block the postsynaptic glutamate response, kynurenic acid was applied just before the physostigmine treatment. fEPSPs for the last 20 min (from 110 min to 130 min) were averaged to examine the physostigmine-induced synaptic suppression (**B**). PPRs were measured during the baseline measurement and at the end of the recording (**C**, **D**). The kynurenic acid pretreatment did not affect the physostigmine-induced presynaptic suppression (**C** and **D**, vehicle, physo: *n* = 5, 6 slices from 3 mice per groups). fEPSPs (**E** and **F**) and PPRs (**G**–**I**) were measured before and after physostigmine perfusion in the Schaffer collateral pathway of the hippocampal slices incubating in ACSF containing 1, 2, or 4 mM Ca^2+^. Averaged depression magnitudes of fEPSPs during the washout period compared to baseline were analyzed (**F**). Among the three Ca^2+^ groups, the 1 mM Ca^2+^ group showed the highest depression magnitude by physostigmine. PPRs were not largely affected in the 1 mM Ca^2+^ group by physostigmine treatment compared to the changed PPRs in the other two groups (**G**–**I**). Graphs represent mean ± S.E.M.; n.s. not significant, physo physostigmine. For detailed statistical information, see Supplementary Table 1.

The physostigmine induced presynaptic suppression could result from either direct presynaptic inhibition or indirect inhibition via retrograde signaling. To investigate these possibilities, we measured fEPSPs and PPRs in the hippocampal Schaffer collateral pathway before and after perfusion of physostigmine in the presence of kynurenic acid, a broad spectrum glutamate receptor antagonist [17]. After washing out kynurenic acid, the physostigmine-treated slices exhibited significantly reduced fEPSPs (Fig. 2A, B) and increased PPRs (Fig. 2C, D). In contrast, slices treated with kynurenic acid alone did not exhibit any changes in fEPSPs and PPRs. These findings show a role for direct presynaptic inhibition in physostigmine-mediated reduced presynaptic glutamate release. We next examined whether indirect inhibition via retrograde signaling changes may also play a role in physostigmine-induced presynaptic suppression. Muscarinic receptor activation can induce synaptic suppression through the production of 2-arachidonoyl glycerol, an innate endocannabinoid, and activate presynaptic CB1 endocannabinoid receptors [18, 19]. The presynaptic CB1 endocannabinoid receptor belongs to the family of G_i/o_ protein-coupled receptors, and its activation leads to a reduction in presynaptic neurotransmitter release by decreasing presynaptic calcium influx and increasing presynaptic potassium channel conductance [20]. We therefore treated slices with AM251, an inverse agonist of the CB1 endocannabinoid receptor, and measured changes in fEPSPs. The administration of AM251 did not affect the physostigmine induced presynaptic suppression, indicating that physostigmine-mediated presynaptic suppression is not triggered by retrograde endocannabinoid signaling (Supplementary Fig. S1A–D). Taken together, these data show physostigmine reduces presynaptic glutamate release via direct presynaptic inhibition.

Neurotransmitter release can be affected by readily releasable pool (RRP) size [21, 22] or presynaptic calcium influx [23]. To elucidate how physostigmine suppresses glutamate release, we first investigated whether physostigmine-induced presynaptic suppression is mediated by a reduction in the presynaptic RRP size. We conducted RRP_train_ analysis by delivering 100 stimulations at a frequency of 20 Hz in the hippocampal Schaffer collateral pathway and plotted cumulative fEPSP slopes against the stimulus number [24]. The intercept of the linear extrapolation on the y-axis, designated as RRP_train_, represents an estimate of RRP size. No significant change was observed in the RRP_train_ with physostigmine, indicating that physostigmine-mediated presynaptic suppression is not induced from alteration in the size of the RRP (Supplementary Fig. S2A, B).

Next, we tested the potential influence of extracellular calcium levels on physostigmine-induced presynaptic suppression. We measured fEPSPs and PPRs in the hippocampal Schaffer collateral pathway using ACSF containing different concentrations of calcium (1 mM, 2 mM, and 4 mM Ca^2+^) before and after physostigmine treatment. In the group exposed to 1 mM Ca^2+^, the magnitude of physostigmine-induced depression was significantly higher compared to the 2 mM and 4 mM Ca^2+^ groups (Fig. 2E, F). Notably, increased PPRs following physostigmine treatment were observed only at an interstimulus interval of 30 msec in the 1 mM Ca^2+^ group, while significantly increased PPRs were observed across broader ranges of inter-stimulus interval conditions in the 2 mM and 4 mM Ca^2+^ concentration groups (Fig. 2G–I). Collectively, these findings suggest that physostigmine induces presynaptic suppression by reducing presynaptic calcium influx from the extracellular medium.

### Physostigmine-induced suppression of presynaptic glutamate release and reduced passive coping behavior are gradually reversible

**Fig. 3:**
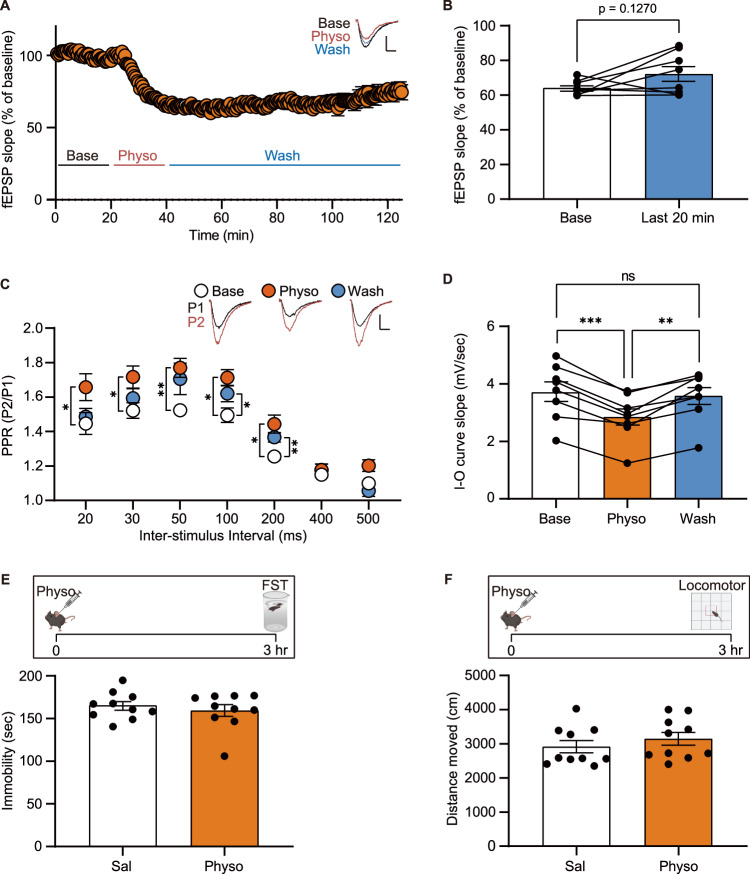
Physostigmine-mediated synaptic and behavioral changes are not persistent. **A**–**D** fEPSPs, input-output (IO) curve slopes, and PPRs were measured before and after physostigmine perfusion in the hippocampal Schaffer collateral pathway. Physostigmine-mediated presynaptic depression gradually dissipated over time (**A**, **B**). The increased PPRs slightly decreased after an 85-min washout period (**C**). In the IO curve analysis, the decreased slope following physostigmine treatment was rescued to basal levels (**D**) (*n* = 8 slices from 4 mice). **E**, **F** The forced swim tests and locomotor activity tests were conducted 3 h after physostigmine treatment (*n* = 10 mice for each group). The physostigmine-treated group did not show any significant change in immobility (**E**) or locomotor activity (**F**) compared to the saline-treated group. Graphs represent mean ± S.E.M.; ns not significant, sal saline, physo physostigmine. For detailed statistical information, see Supplementary Table 1.

Clinical studies have reported that depressive symptoms induced by physostigmine present within 20 min and are resolved within one hour, suggesting the effects of physostigmine are transient [4, 5, 25]. To establish a correlation between these clinical findings and physostigmine-induced synaptic suppression, we measured fEPSP and PPRs in the hippocampal Schaffer collateral pathway for an hour following a 20-min perfusion of physostigmine. In 5 out of 8 slices, the suppressed synaptic response began to recover after 85 min (Fig. 3A, B). Moreover, the increased PPRs at interstimulus intervals of 100 and 200 msec as well as the decreased slopes of the input-output curves following physostigmine treatment returned to basal levels after 85 min of washout (Fig. 3C, D).

**Fig. 4:**
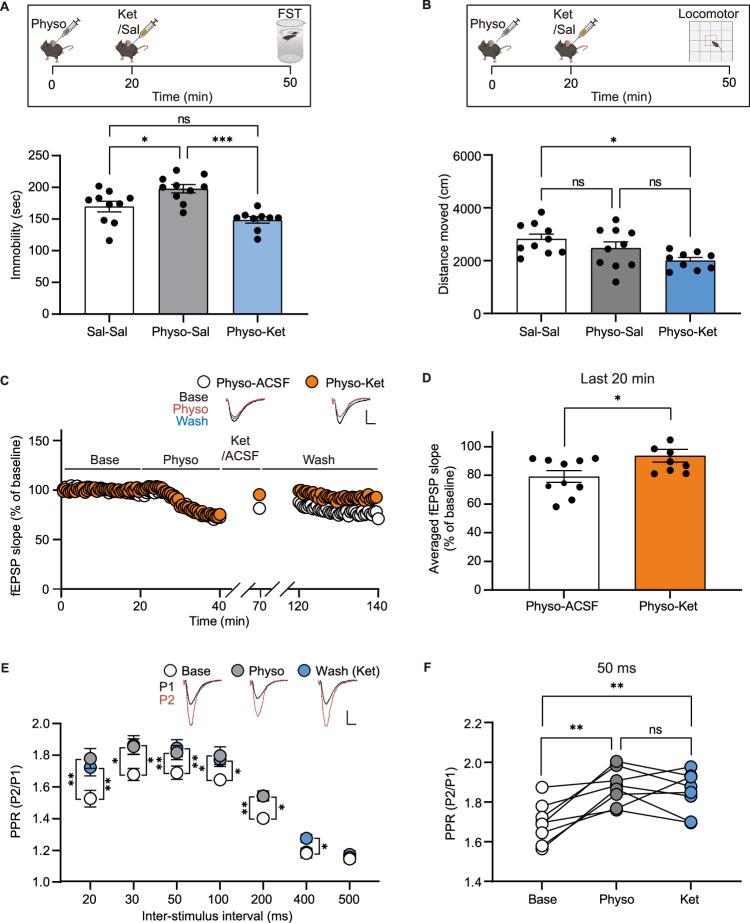
Ketamine relieves physostigmine-induced presynaptic suppression and changes in passive coping behavior. **A**, **B** Ketamine was given 20 min after physostigmine treatment, and then the forced swim test or locomotor activity test was conducted 30 min after the ketamine treatment [*n* = 10 (Sal-Sal), 10 (Physo-Sal), and 9 (Physo-Ket) mice]. Physostigmine significantly increased immobile duration, whereas the change was not observed in the physostigmine with ketamine-treated group (**A**). Physostigmine followed by ketamine significantly reduced locomotor activity compared to the control group (**B**). **C**–**F** fEPSPs and PPRs were measured before and after perfusion of physostigmine and ketamine in the hippocampal Schaffer collateral pathway (*n* = 10 (physo-ACSF) and 8 (physo-ket) slices from 5 and 4 mice, repectively). Ketamine restored the suppressed synaptic activity following physostigmine treatment to basal levels (**C**, **D**). The increased PPRs following physostigmine treatment were not significantly changed by ketamine (**E**, **F**). Graphs represent mean ± S.E.M.; ns not significant, sal saline, physo physostigmine. For detailed statistical information, see Supplementary Table 1.

We observed that physostigmine significantly increases immobility in the forced swim test (FST) at 50 min post-injection (see Fig. 4A). Given the transient nature of physostigmine to induce depressive symptoms in patients, we examined whether the behavioral effects were also transient in mice. Indeed, we observed no difference in immobility duration between the physostigmine-treated and vehicle control group 3 h post-injection (Fig. 3E). We also observed no change in locomotor activity as monitored for 20 min at 3 h post physostigmine treatment (Fig. 3F). Thus, these results suggest that the physostigmine-induced hippocampal presynaptic suppression which gradually recovers after 85 min, may contribute to the dissipation of change in floating behavior in the FST.

Next, we aimed to better delineate the relationship between physostigmine-induced presynaptic suppression and the passive coping behavior as assessed by the FST. Previous data has shown that the anticholinergic drug scopolamine, a muscarinic antagonist, blocks physostigmine-induced depression-like behaviors [7]. Therefore, we examined whether scopolamine prevents the physostigmine induced presynaptic suppression. Indeed, we observed scopolamine prevented the increase in PPRs following physostigmine treatment (Supplementary Fig. S3A, B). These data suggest that physostigmine induces rapid and transient presynaptic suppression of glutamate release through alterations in presynaptic muscarinic receptor signaling. Taken together, scopolamine may prevent physostigmine-mediated acute stress by relieving the suppressed hippocampal presynaptic glutamate release and providing further support for the specific change in presynaptic suppression producing the changes in behavior [7, 12].

### Ketamine rescues physostigmine-mediated reduction in passive coping behavior and hippocampal synaptic depression

Ketamine is a NMDA receptor antagonist that produces rapid antidepressant action. We examined whether ketamine reverses the physostigmine induced behaviors as assessed in the FST (Fig. 4A). Mice were treated with physostigmine, followed by ketamine administration 20 min later, and the FST conducted 30 min after the ketamine injection. Mice treated with physostigmine alone displayed a significant increase in immobility, suggestive of a reduced passive coping response. We observed that ketamine treatment significantly reduced the heightened immobility produced by physostigmine suggesting it rescued the behavioral deficit. We examined the mice for changes in locomotor (Fig. 4B). Mice injected with physostigmine alone did not have changes in locomotor activity, although there was a decrease in activity in mice administered physostigmine and ketamine. However, a decrease in locomotor activity would not account for the reduced immobility observed in the FST. Our findings that ketamine reverses physostigmine induced behavioral effects may not necessarily be surprising given that ketamine produces antidepressant action in depressed [26] and treatment resistant depressed patients [27].

The rapid antidepressant action of ketamine has been linked to its ability to promote synaptic potentiation in the hippocampus by blocking NMDA receptors leading to a rapid increase in BDNF protein, which activates tropomyosin receptor kinase B (TrkB) resulting in increased AMPA receptors on the postsynaptic surface of the hippocampal Schaffer collateral pathway [28, 29]. Recent work has shown BDNF-TrkB signaling postsynaptically at CA3-CA1 synapses is required for ketamine’s synaptic and behavioral action [30]. We therefore assessed whether physostigmine-induced presynaptic suppression requires TrkB signaling using conditional TrkB knockout mice in which the receptor is postnatally deleted in excitatory neurons in the forebrain. We observed that physostigmine perfusion resulted in similar decreased fEPSPs and increased PPRs in control littermates and conditional *Trkb* knockout mice, demonstrating that intracellular BDNF-TrkB signaling in excitatory neurons is not essential to the physostigmine-mediated synaptic suppression (Supplementary Fig. S4A–D).

While physostigmine does not require TrkB signaling to impair presynaptic glutamate release, the question remains whether this synaptic deficit is rescued by ketamine. Ketamine’s antidepressant action has been linked to its ability to augment fEPSPs in the hippocampal Schaffer collateral pathway [12, 28]. Therefore, we perfused physostigmine onto hippocampal slices followed by ketamine perfusion and then measured fEPSPs and PPRs before and after the treatments. We found that ketamine restored the physostigmine mediated suppressed synaptic activity almost to basal levels. However, the increased PPRs following physostigmine treatment were not significantly altered by ketamine treatment showing the impairment in presynaptic glutamate release remained (Fig. 4C–F). Thus, ketamine did not rectify the presynaptic suppression induced by physostigmine, but rather it counterbalanced the suppressed hippocampal synaptic activity through ketamine-mediated postsynaptic potentiation.

## Discussion

In the current study, we took advantage of physostigmine’s ability to transiently and acutely induce stress and examined its impact on synaptic transmission and whether the rapid antidepressant action of ketamine corrects these synaptic deficits. We observed that physostigmine induces presynaptic suppression of neurotransmitter release via a Ca^2+^-dependent and muscarinic receptor mediated mechanism in the hippocampal Schaffer collateral pathway consistent with its time course to induce acute stress. Ketamine rapidly reversed the physostigmine-mediated behavioral effects but did not correct the deficits in presynaptic neurotransmitter release probability. Rather, ketamine acted via a postsynaptic mechanism to offset the impaired synaptic activity due to the suppression of presynaptic neurotransmitter release. Taken together, these data demonstrate that physostigmine produces specific presynaptic effects on synaptic transmission, and while ketamine rescues the behavioral effects, it does not correct these specific synaptic alterations but rather produces rapid synaptic effects that ‘mask’ or ‘counterbalance’ the suppressed activity.

Stress is often used to model depressive-related behavior. Some stress paradigms have been shown to elevate hippocampal acetylcholine levels and activate the septo-hippocampal cholinergic system [31]. Clinical Single Photon Emission Computed Tomography (SPECT) imaging has demonstrated an increase in acetylcholine concentrations across various brain regions in patients with unipolar and bipolar depression [32]. Similarly, in murine models, hippocampal acetylcholine levels rise during or after stressors like foot shock and restraint [8, 33–35]. Prior research has reported that enhancing cholinergic signaling in the hippocampus—either through acetylcholinesterase deletion or through chemogenetic/optogenetic induction of acetylcholine release—elicits depression-like behaviors and increases stress susceptibility in mice [7, 8]. However, the precise mechanisms by which elevated acetylcholine mediates these behavioral changes remain elusive. A key consideration is that the type and duration of stress can produce differing physiological and behavioral effects. Therefore, we used physostigmine, which produces a transient period of depressed mood in humans, to understand the synaptic alterations that mediate the behavior.

Our investigations with physostigmine suggest that increased cholinergic activity leads to hippocampal synaptic suppression, which in turn could be a key factor in stress-related behaviors. This hypothesis is supported by correlations found in previous and current studies between mood behavioral changes and hippocampal presynaptic inhibition. Notably, recent in vivo calcium imaging studies reveal that physostigmine significantly amplifies the prevalence of hypoactive cells in the hippocampal CA1 region at the same dosage (0.25 mg/kg) used in our behavior experiments [36]. We also observed that both hippocampal presynaptic suppression and behavioral effects following physostigmine shows a strong correlation. Physostigmine-induced acute stress and the suppression of glutamatergic release were reversible by scopolamine pre-treatment (Supplementary Fig. S3) and in earlier studies [7, 8]. Post-physostigmine washout, fEPSPs gradually returned to baseline levels, consistent with the observed behavioral changes (Fig. 3).

In the synapse, physostigmine increases levels of acetylcholine by preventing its degradation. Elevated levels of acetylcholine possibly activate presynaptic auto-receptors which are associated with G_i/o_ proteins. Upon activation of the G_i/o_-coupled receptor, the Gβγ subunit is released, leading to the inhibition of presynaptic calcium channels (CaV2 channels). This inhibition, in turn, results in the reduced influx of extracellular Ca^2+^ [37–39] which attenuates exocytosis of synaptic vesicles [23, 40]. While this model starts to provide a framework to our current findings, the potential role of auto-receptors in physostigmine-mediated effects requires further investigation.

Ketamine produces rapid antidepressant action that has been hypothesized to be due to NMDA receptor mediated BDNF-TrkB intracellular signaling that triggers fEPSP potentiation at Schaffer collateral synapses [12, 28, 41]. Ketamine blocks NMDA receptors at rest and inhibits the influx of Ca^2+^. This inhibition subsequently reduces the activity of Ca^2+^-dependent eEF2 kinase (eEF2K). Decreased eEF2K activity leads to reduced phosphorylation of its substrate, eukaryotic elongation factor 2 (eEF2). eEF2 is a translation elongation factor and the reduction in phosphorylation facilitates the translation of brain-derived neurotrophic factor (BDNF), a key protein in synaptic plasticity. Increased BDNF activates TrkB receptor and its downstream signaling to promote the synaptic incorporation of AMPA receptors in hippocampal CA1 area [28–30, 41–44]. The localized deletion of TrkB at CA1 (postsynaptic), but not CA3 (presynaptic), synapses has been shown to be sufficient to occlude the antidepressant and synaptic action of ketamine and substantiate the postsynaptic site of action [30]. We observed that physostigmine decreases fEPSPs and decreases the probability of neurotransmitter release in conditional *Trkb* knockout mice similar to wildtype mice showing that TrkB signaling is not required for the synaptic suppression. Moreover, the physostigmine-induced depressive phenotype is not interacting with key molecular determinants of ketamine’s antidepressant action.

Ketamine produces rapid antidepressant effects in patients with MDD and treatment-resistant depression. Therefore, our finding that acute ketamine administration to mice treated with physostigmine rescued the behavioral effects is not unexpected. Our focus on examining the synaptic deficits of physostigmine induced depression and whether ketamine rescues these deficits provides a novel opportunity to assess antidepressant action. Given the physostigmine-induced deficits and the ketamine-mediated synaptic plasticity that are suggestive of driving the behavior, we examined fEPSPs and release probability in hippocampal slices to test our hypothesis. We observed that ketamine restored the physostigmine induced fEPSPs to baseline levels but did not rescue the changes in probability of glutamate release probability, leaving the inherent synaptic deficits intact. These data further substantiate the postsynaptic action of ketamine and show it can offset impaired synaptic activity resulting from presynaptic mechanisms although it does not correct the actual synaptic deficits. Indeed, the rapid antidepressant action of ketamine in treatment resistant depression patients, which may have been afflicted by the disorder for years, suggests functional synaptic changes may be a critical component of the response rather than ‘fixing’ years of pathology in a time frame of a few hours. Therefore, patients initially responding to ketamine treatment may still possess the synaptic deficits that underlie depression. These findings highlight key principles underlying rapid antidepressant action as well as pinpoint critical differences between the mechanisms that underlie depression versus antidepressant action.

## Supplementary information

Supplementary Figures

Supplementary Table

## Data Availability

The datasets from this paper are presented in the supporting files.

## References

[1] Jesulola E, Micalos P, Baguley IJ. Understanding the pathophysiology of depression: From monoamines to the neurogenesis hypothesis model - are we there yet? Behav Brain Res. 2018;341:79–90. 29284108 10.1016/j.bbr.2017.12.025

[2] Kim JW, Suzuki K, Kavalali ET, Monteggia LM. Ketamine: Mechanisms and Relevance to Treatment of Depression. Annu Rev Med. 2023;75:129–43. 37729028 10.1146/annurev-med-051322-120608

[3] Abdallah CG, Sanacora G, Duman RS, Krystal JH. Ketamine and rapid-acting antidepressants: a window into a new neurobiology for mood disorder therapeutics. Annu Rev Med. 2015;66:509–23. 25341010 10.1146/annurev-med-053013-062946 PMC4428310

[4] Risch SC, Cohen RM, Janowsky DS, Kalin NH, Sitaram N, Gillin JC, et al. Physostigmine induction of depressive symptomatology in normal human subjects. Psychiatry Res. 1981;4:89–94. 7012883 10.1016/0165-1781(81)90012-3

[5] Janowsky DS, el-Yousef K, Davis JM, Sekerke HJ. Parasympathetic suppression of manic symptoms by physostigmine. Arch Gen Psychiatry. 1973;28:542–7. 4692153 10.1001/archpsyc.1973.01750340072012

[6] Dulawa SC, Janowsky DS. Cholinergic regulation of mood: from basic and clinical studies to emerging therapeutics. Mol Psychiatry. 2019;24:694–709. 30120418 10.1038/s41380-018-0219-x PMC7192315

[7] Mineur YS, Obayemi A, Wigestrand MB, Fote GM, Calarco CA, Li AM, et al. Cholinergic signaling in the hippocampus regulates social stress resilience and anxiety- and depression-like behavior. Proc Natl Acad Sci USA. 2013;110:3573–8. 23401542 10.1073/pnas.1219731110 PMC3587265

[8] Mineur YS, Mose TN, Vanopdenbosch L, Etherington IM, Ogbejesi C, Islam A, et al. Hippocampal acetylcholine modulates stress-related behaviors independent of specific cholinergic inputs. Mol Psychiatry. 2022;27:1829–38. 34997190 10.1038/s41380-021-01404-7 PMC9106825

[9] Luikart BW, Nef S, Virmani T, Lush ME, Liu Y, Kavalali ET, et al. TrkB has a cell-autonomous role in the establishment of hippocampal Schaffer collateral synapses. J Neurosci. 2005;25:3774–86. 15829629 10.1523/JNEUROSCI.0041-05.2005 PMC6724922

[10] Chen RZ, Akbarian S, Tudor M, Jaenisch R. Deficiency of methyl-CpG binding protein-2 in CNS neurons results in a Rett-like phenotype in mice. Nat Genet. 2001;27:327–31. 11242118 10.1038/85906

[11] Kim JW, Herz J, Kavalali ET, Monteggia LM. A key requirement for synaptic Reelin signaling in ketamine-mediated behavioral and synaptic action. Proc Natl Acad Sci USA. 2021;118:e2103079118. 10.1073/pnas.2103079118 PMC8157952 33975959

[12] Kim JW, Autry AE, Na ES, Adachi M, Bjorkholm C, Kavalali ET, et al. Sustained effects of rapidly acting antidepressants require BDNF-dependent MeCP2 phosphorylation. Nat Neurosci. 2021;24:1100–09. 34183865 10.1038/s41593-021-00868-8 PMC8338784

[13] Kim JW, Monteggia LM. Increasing doses of ketamine curtail antidepressant responses and suppress associated synaptic signaling pathways. Behav Brain Res. 2020;380:112378. 31760154 10.1016/j.bbr.2019.112378 PMC7136035

[14] Dobrunz LE, Stevens CF. Heterogeneity of release probability, facilitation, and depletion at central synapses. Neuron. 1997;18:995–1008. 9208866 10.1016/s0896-6273(00)80338-4

[15] Mans RA, Warmus BA, Smith CC, McMahon LL. An acetylcholinesterase inhibitor, eserine, induces long-term depression at CA3-CA1 synapses in the hippocampus of adult rats. J Neurophysiol. 2014;112:2388–97. 25143547 10.1152/jn.00048.2014 PMC4315450

[16] Rosenmund C, Clements JD, Westbrook GL. Nonuniform probability of glutamate release at a hippocampal synapse. Science. 1993;262:754–7. 7901909 10.1126/science.7901909

[17] Salin PA, Malenka RC, Nicoll RA. Cyclic AMP mediates a presynaptic form of LTP at cerebellar parallel fiber synapses. Neuron. 1996;16:797–803. 8607997 10.1016/s0896-6273(00)80099-9

[18] Kim J, Isokawa M, Ledent C, Alger BE. Activation of muscarinic acetylcholine receptors enhances the release of endogenous cannabinoids in the hippocampus. J Neurosci. 2002;22:10182–91. 12451119 10.1523/JNEUROSCI.22-23-10182.2002 PMC6758770

[19] Martin HG, Bernabeu A, Lassalle O, Bouille C, Beurrier C, Pelissier-Alicot AL, et al. Endocannabinoids Mediate Muscarinic Acetylcholine Receptor-Dependent Long-Term Depression in the Adult Medial Prefrontal Cortex. Front Cell Neurosci. 2015;9:457. 26648844 10.3389/fncel.2015.00457 PMC4664641

[20] Chevaleyre V, Takahashi KA, Castillo PE. Endocannabinoid-mediated synaptic plasticity in the CNS. Annu Rev Neurosci. 2006;29:37–76. 16776579 10.1146/annurev.neuro.29.051605.112834

[21] Stanton PK, Winterer J, Bailey CP, Kyrozis A, Raginov I, Laube G, et al. Long-term depression of presynaptic release from the readily releasable vesicle pool induced by NMDA receptor-dependent retrograde nitric oxide. J Neurosci. 2003;23:5936–44. 12843298 10.1523/JNEUROSCI.23-13-05936.2003 PMC6741233

[22] Goda Y, Stevens CF. Readily releasable pool size changes associated with long term depression. Proc Natl Acad Sci USA. 1998;95:1283–8. 9448323 10.1073/pnas.95.3.1283 PMC18746

[23] Dolphin AC, Lee A. Presynaptic calcium channels: specialized control of synaptic neurotransmitter release. Nat Rev Neurosci. 2020;21:213–29. 32161339 10.1038/s41583-020-0278-2 PMC7873717

[24] Kaeser PS, Regehr WG. The readily releasable pool of synaptic vesicles. Curr Opin Neurobiol. 2017;43:63–70. 28103533 10.1016/j.conb.2016.12.012 PMC5447466

[25] Oppenheim G, Ebstein RP, Belmaker RH. Effect of lithium on the physostigmine-induced behavioral syndrome and plasma cyclic GMP. J Psychiatr Res. 1979;15:133–8. 40018 10.1016/0022-3956(79)90024-4

[26] Berman RM, Cappiello A, Anand A, Oren DA, Heninger GR, Charney DS, et al. Antidepressant effects of ketamine in depressed patients. Biol Psychiatry. 2000;47:351–4. 10686270 10.1016/s0006-3223(99)00230-9

[27] Zarate CA Jr., Singh JB, Carlson PJ, Brutsche NE, Ameli R, Luckenbaugh DA, et al. A randomized trial of an N-methyl-D-aspartate antagonist in treatment-resistant major depression. Arch Gen Psychiatry. 2006;63:856–64. 16894061 10.1001/archpsyc.63.8.856

[28] Autry AE, Adachi M, Nosyreva E, Na ES, Los MF, Cheng PF, et al. NMDA receptor blockade at rest triggers rapid behavioural antidepressant responses. Nature. 2011;475:91–5. 21677641 10.1038/nature10130 PMC3172695

[29] Kim JW, Suzuki K, Kavalali ET, Monteggia LM. Bridging rapid and sustained antidepressant effects of ketamine. Trends Mol Med. 2023;29:364–75. 36907686 10.1016/j.molmed.2023.02.003 PMC10101916

[30] Lin PY, Ma ZZ, Mahgoub M, Kavalali ET, Monteggia LM. A synaptic locus for TrkB signaling underlying ketamine rapid antidepressant action. Cell Rep. 2021;36:109513. 34407417 10.1016/j.celrep.2021.109513 PMC8404212

[31] Kniffin A, Bangasser DA, Parikh V. Septohippocampal cholinergic system at the intersection of stress and cognition: Current trends and translational implications. Eur J Neurosci. 2024;59:2155–80. 10.1111/ejn.15999 PMC10875782 37118907

[32] Saricicek A, Esterlis I, Maloney KH, Mineur YS, Ruf BM, Muralidharan A, et al. Persistent beta2*-nicotinic acetylcholinergic receptor dysfunction in major depressive disorder. Am J Psychiatry. 2012;169:851–9. 22772158 10.1176/appi.ajp.2012.11101546 PMC3494404

[33] Mark GP, Rada PV, Shors TJ. Inescapable stress enhances extracellular acetylcholine in the rat hippocampus and prefrontal cortex but not the nucleus accumbens or amygdala. Neuroscience. 1996;74:767–74. 8884772 10.1016/0306-4522(96)00211-4

[34] Finkelstein Y, Koffler B, Rabey JM, Gilad GM. Dynamics of cholinergic synaptic mechanisms in rat hippocampus after stress. Brain Res. 1985;343:314–9. 4052753 10.1016/0006-8993(85)90749-8

[35] Tajima T, Endo H, Suzuki Y, Ikari H, Gotoh M, Iguchi A. Immobilization stress-induced increase of hippocampal acetylcholine and of plasma epinephrine, norepinephrine and glucose in rats. Brain Res. 1996;720:155–8. 8782908 10.1016/0006-8993(96)00046-7

[36] Zhou H, Li H, Gowravaram N, Quan M, Kausar N, Gomperts SN. Disruption of hippocampal neuronal circuit function depends upon behavioral state in the APP/PS1 mouse model of Alzheimer’s disease. Sci Rep. 2022;12:21022. 36471155 10.1038/s41598-022-25364-2 PMC9723144

[37] Khaziev E, Samigullin D, Zhilyakov N, Fatikhov N, Bukharaeva E, Verkhratsky A, et al. Acetylcholine-Induced Inhibition of Presynaptic Calcium Signals and Transmitter Release in the Frog Neuromuscular Junction. Front Physiol. 2016;7:621. 28018246 10.3389/fphys.2016.00621 PMC5149534

[38] Allen TG, Abogadie FC, Brown DA. Simultaneous release of glutamate and acetylcholine from single magnocellular “cholinergic” basal forebrain neurons. J Neurosci. 2006;26:1588–95. 16452682 10.1523/JNEUROSCI.3979-05.2006 PMC6675485

[39] Dolezal V, Tucek S. Calcium channels involved in the inhibition of acetylcholine release by presynaptic muscarinic receptors in rat striatum. Br J Pharm. 1999;127:1627–32. 10.1038/sj.bjp.0702721 PMC1566163 10455319

[40] Blackmer T, Larsen EC, Takahashi M, Martin TF, Alford S, Hamm HE. G protein betagamma subunit-mediated presynaptic inhibition: regulation of exocytotic fusion downstream of Ca2+ entry. Science. 2001;292:293–7. 11303105 10.1126/science.1058803

[41] Nosyreva E, Szabla K, Autry AE, Ryazanov AG, Monteggia LM, Kavalali ET. Acute suppression of spontaneous neurotransmission drives synaptic potentiation. J Neurosci. 2013;33:6990–7002. 23595756 10.1523/JNEUROSCI.4998-12.2013 PMC3661220

[42] Suzuki K, Kim JW, Nosyreva E, Kavalali ET, Monteggia LM. Convergence of distinct signaling pathways on synaptic scaling to trigger rapid antidepressant action. Cell Rep. 2021;37:109918. 34731624 10.1016/j.celrep.2021.109918 PMC8590465

[43] Gideons ES, Kavalali ET, Monteggia LM. Mechanisms underlying differential effectiveness of memantine and ketamine in rapid antidepressant responses. Proc Natl Acad Sci USA. 2014;111:8649–54. 24912158 10.1073/pnas.1323920111 PMC4060670

[44] Kavalali ET, Monteggia LM. Rapid homeostatic plasticity and neuropsychiatric therapeutics. Neuropsychopharmacology. 2023;48:54–60. 35995973 10.1038/s41386-022-01411-4 PMC9700859

