# Supplementary material for: Distinct synaptic mechanisms drive the behavioral response to acute stress and rapid correction by ketamine
Source: Neuropsychopharmacology. 2024 Jul 1;49(12):1916–24. doi: 10.1038/s41386-024-01908-0 (PMC11473657; doi:10.1038/s41386-024-01908-0)
Supplement: Supplementary file 1 — Supplementary Figures [file 41386_2024_1908_MOESM1_ESM.pdf]

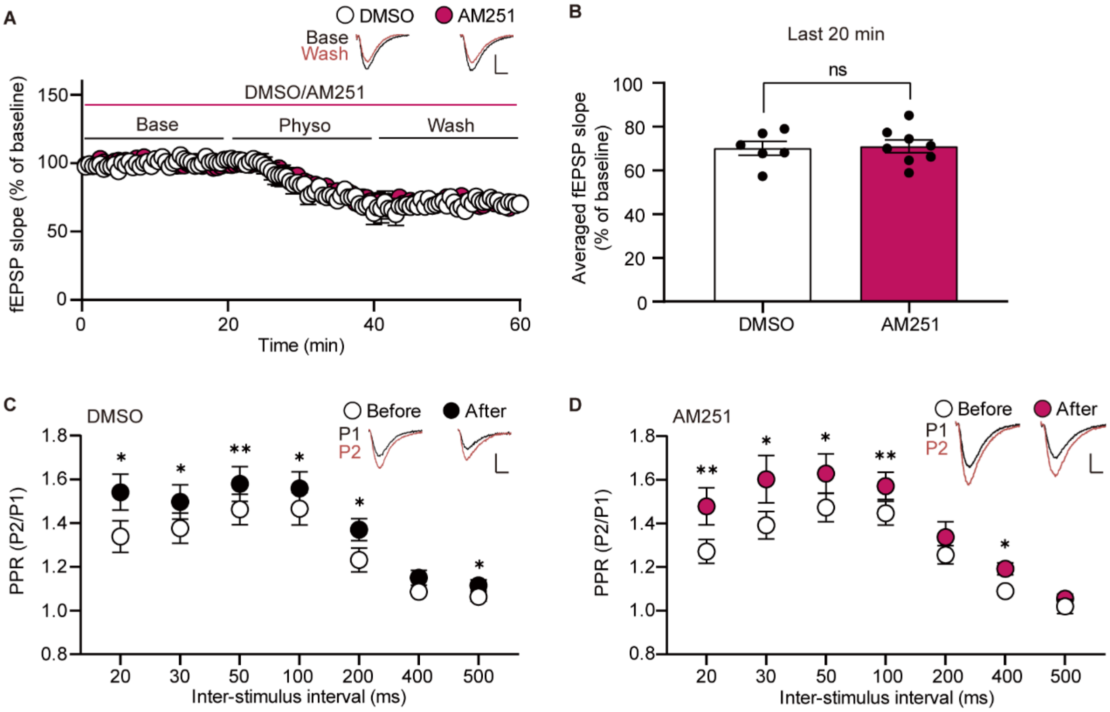

Fig. S1. Endocannabinoid signaling does not affect physostigmine-induced presynaptic depression.

(A-D) fEPSPs and PPRs were measured before and after physostigmine perfusion in the hippocampal Schaffer collateral pathway, and the AM251, eCB1 reverse agonist, was applied whole during the recording (n = 6 (DMSO) and 8 (AM251) slices). The inverse agonism of eCB1 did not affect physostigmine-induced presynaptic suppression. Graphs represent mean ± S.E.M.; n.s.: not significant, *, P < 0.05, **, P < 0.01, Physo: physostigmine. For detailed statistical information, see Supplementary Table 1.

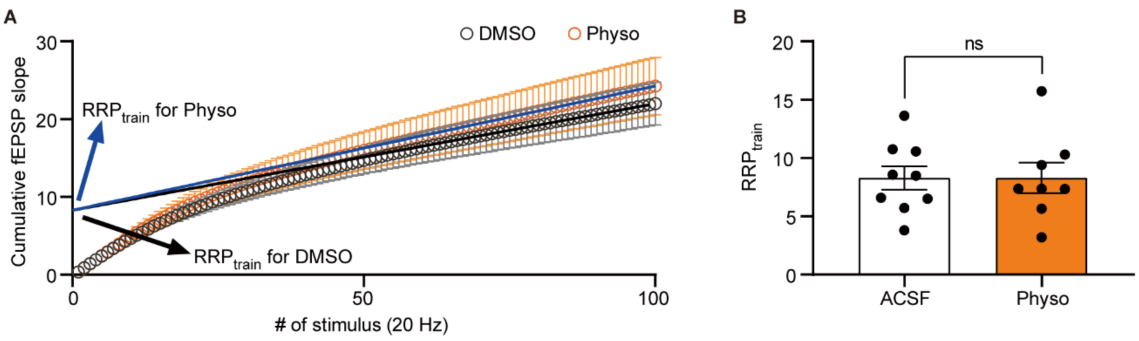

Fig. S2. Physostigmine does not affect the size of readily releasable pool.

(A and B) fEPSPs slopes were measured during the delivery of 100 stimulation at 20 Hz in the hippocampal Schaffer collateral pathway. The cumulative fEPSPs slope were plotted and linearly extrapolated to calculate the intercept of y-axis with the last 25 plotted cumulative fEPSPs slopes (n = 9 (ACSF) and 10 (physo) slices). The intercept of y-axis designated as RRP_{train} (A). No change in RRP_{train} was observed following physostigmine treatment (B). Graphs represent mean ± S.E.M.; n.s.: not significant, ACSF: artificial cerebrospinal fluid, Physo: physostigmine. For detailed statistical information, see Supplementary Table 1.

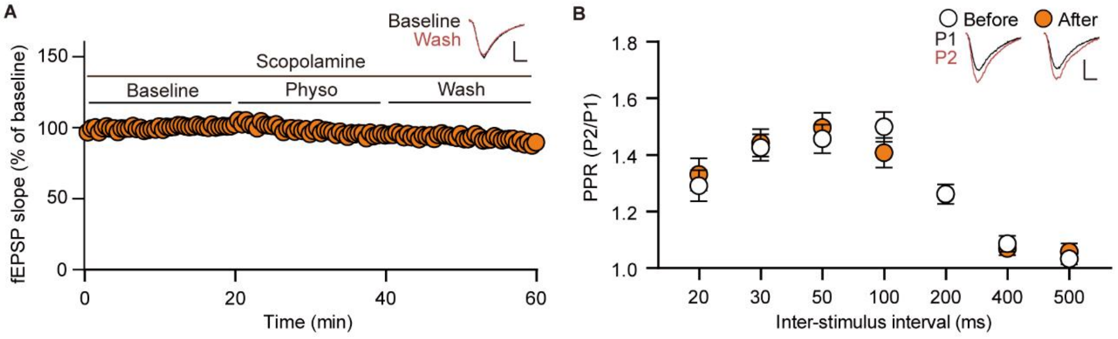

Fig. S3. Muscarinic receptor blockade by scopolamine prevents physostigmine-mediated presynaptic suppression.

(A and B) fEPSPs and PPRs were measured before and after physostigmine under the application of scopolamine in the hippocampal Schaffer collateral pathway. Scopolamine prevented physostigmine-induced suppression of synaptic response (A) and PPRs (B) (n = 10 slices). Graphs represent mean ± S.E.M.; N.S.: not significant, *, P < 0.05, **, P < 0.01, ***, P < 0.001. ACSF: artificial cerebrospinal fluid, physo: physostigmine. For detailed statistical information, see Supplementary Table 1.

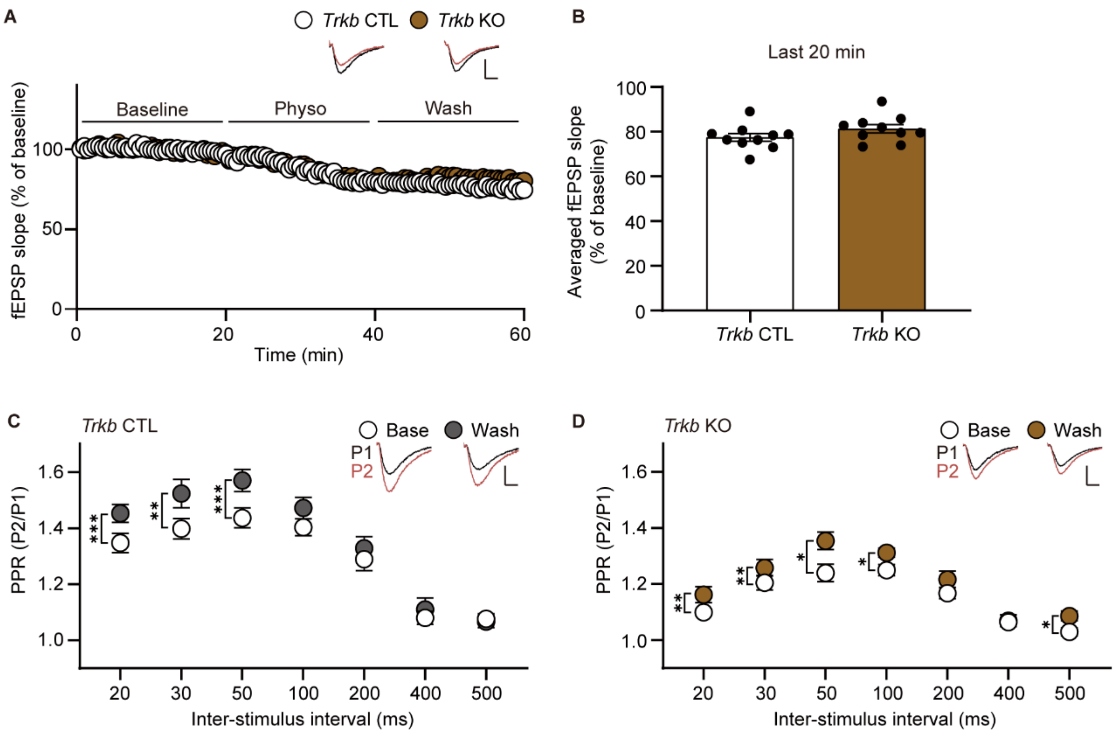

Fig. S4. Deletion of TrkB does not affect the physostigmine-mediated presynaptic suppression.

(A-D) fEPSPs and PPRs were measured before and after physostigmine in the hippocampal Schaffer collateral pathway of Trkb CTL and KO mice (n = 10 slices for each group). Physostigmine produces presynaptic suppression in the Trkb CTL and KO mice. CTL: Trkb littermate control mice, KO: Trkb conditional knockout mice. Graphs represent mean ± S.E.M.; ns: not significant, Physo: physostigmine. For detailed statistical information, see Supplementary Table 1.
